# Precipitation-driven restructuring of rhizosphere microbiota enhances alpine plant adaptation

**DOI:** 10.3389/fpls.2025.1641511

**Published:** 2025-10-08

**Authors:** Chao Chen, Dafeng Xu, Benli Jiang, Xianyong Lu, Chun Yu, Yujiao Wang, Hongjuan Wang, Jingna Li, Jiabao Zhu

**Affiliations:** ^1^ Institute of Industrial Crops, Anhui Academy of Agricultural Sciences, Hefei, China; ^2^ School of Biological and Food Engineering, Hefei Normal University, Hefei, China

**Keywords:** *Poa alpigena*, precipitation, rhizosphere microbiomes, alpine sandy ecosystems, microbial adaptation

## Abstract

**Introduction:**

Climate-driven precipitation changes are increasingly threatening alpine ecosystems, yet the adaptive responses of soil microbiomes to rainfall variability remain poorly characterized. This knowledge gaphinders our ability to predict ecosystem resilience under future climate scenarios.

**Methods:**

We combined metagenomic sequencing with detailed physicochemical analyses to examine how natural precipitation events reshape the microbial communities in both rhizosphere and bulk soils associated with *Poa alpigena* in the alpine sandy ecosystems of Qinghai Lake.

**Results:**

Rainfall significantly reduced bacterial alpha diversity, particularly in bulk soils, and triggered a compositional shift from drought-resistant taxa (e.g., *Geobacter*, *Pseudomonas*) to moisture-adapted genera (e.g., *Azospirillum*, *Methylobacterium*). Actinobacteria remained consistently dominant (31.56-34.62%), while *Proteobacteria* abundance decreased markedly in the rhizosphere post-rainfall. Metabolic reconstruction revealed a transition from pre-rainfall carbohydrate catabolism to post-rainfall anaerobic energy production and carbon fixation pathways. The rhizosphere microbiome uniquely displayed drought-induced biofilm formation and rainfall-enhanced branched-chain amino acid metabolism. Soil moisture and total carbon were identified as primary drivers of microbial restructuring in bulk soils, whereas root exudates conferred stability to rhizosphere communities against hydrological fluctuations.

**Discussion:**

These results elucidate microbiome-mediated adaptive strategies to precipitation changes in alpine sandy ecosystems, highlighting the critical buffering role of plant-microbe interactions. The study provides a mechanistic basis for predicting and restoring climatevulnerable wetlands under increasingly variable hydrological regimes.

## 1 Introduction

Located on the northeastern Qinghai-Tibet Plateau, the Qinghai Lake Basin represents a critical ecological transition zone featuring characteristic alpine wetland ecosystems with vital barrier functions (Hou et al., 2020). This globally significant climate-sensitive region exhibits a distinctive yet fragile ecosystem pattern shaped by its unique high-altitude environment and semi-arid climate. The basin’s ecological processes are profoundly influenced by spatiotemporal variations in precipitation, which directly regulate rhizospheric moisture dynamics and associated microbial responses (Yang et al., 2021), while simultaneously affecting plant community biomass and coverage to reshape soil microbial composition. The root-soil interface serves as a vital ecological nexus where rhizosphere microbial communities mediate plant-soil interactions through multiple processes including nutrient solubilization, nitrogen fixation, and metabolic exchanges, thereby enhancing plant nutrient acquisition and environmental stress resilience (Amine et al., 2018). Notably, rhizosphere microbial assemblages display remarkable variability in composition, abundance and diversity, attributable to interspecific differences in plant genetics, root exudate profiles and root architecture (Smilauer et al., 2020). Functioning as dynamic plant-soil intermediaries, these microbial communities maintain ecosystem stability by orchestrating key processes such as nutrient cycling, water use optimization and stress resistance enhancement (Wan et al., 2024), making them central to ecological responses following precipitation pulses.

*Poa alpigena* Lindm is regarded as a typical model forage grass due to its remarkable adaptability to alpine habitats, robust regenerative capacity, and excellent forage quality. Its low-growing stature and well-developed root system enable it to effectively tolerate environmental stresses such as low temperatures, strong radiation, and short growing seasons, making it an ideal subject for studying stress physiology and ecological adaptation in forage grasses. Furthermore, its high nutritional value, good palatability, and outstanding grazing tolerance play a crucial role in ensuring the stability of animal husbandry in alpine regions. It also serves as a key species for research on sustainable grassland productivity, ecological restoration, and grazing management strategies (Fan et al., 2018). Notably, following precipitation events, its rhizosphere microenvironment undergoes rapid physicochemical changes that subsequently drive restructuring of microbial community composition and functionality (Brokate et al., 2024). This dynamic response process directly influences the plant’s water-use efficiency and environmental adaptability (Heffernan and Fisher, 2012). This process represents a critical component in understanding the restoration mechanisms of alpine sandy ecosystems. For instance, Liu et al. (2023) revealed that water availability supersedes plant species in regulating microbial functional genes in Tibetan alpine grasslands, where increased precipitation boosted microbial gene abundance while soil C/N/K nutrients showed negative correlations with functional potential, offering new perspectives on microbial drought adaptation in semi-arid ecosystems.

High-throughput next-generation sequencing (NGS) has revolutionized metagenomic investigations by providing unprecedented sequencing depth while dramatically reducing per-base costs (Masuda et al., 2024). Metagenomic approaches, which involve direct extraction and sequencing of total environmental DNA, circumvent the well-documented limitations of culture-dependent methods, permitting exhaustive characterization of microbial community composition, phylogenetic architecture, and functional potential at an ecosystem scale (Parmar et al., 2024). This culture-independent paradigm has proven indispensable for probing the “microbial dark matter” that dominates most environments, driving transformative advances in both environmental and medical microbiology (Trivett et al., 2025).

This study employs shotgun metagenomics to investigate precipitation-driven dynamics in Poa alpigena rhizosphere microbiomes within Qinghai Lake’s Bird Island sand dunes, conducting temporally resolved comparative analyses of pre- and post-precipitation periods to characterize microbial functional gene repertoire shifts and assess their bioremediation potential. By integrating soil physicochemical data with microbial community profiles, we identify key environmental drivers of assemblage dynamics while elucidating successional patterns and functional reorganization following water input, thereby deciphering plant-microbe co-adaptation mechanisms to alpine moisture fluctuations. These findings not only advance theoretical frameworks for alpine biogeochemical cycling but also provide practical solutions through identification of functional microbial taxa and metabolic pathways applicable to degraded grassland restoration, ultimately establishing a mechanistic basis for conservation strategies targeting ecosystem resilience and sustainable management while enhancing regional ecological security.

## 2 Materials and methods

### 2.1 Study area

The study was conducted in the Qinghai Lake Bird Island National Nature Reserve (36°57’-37°04′N, 99°44’-99°54′E), located in the northwestern part of Qinghai Lake at an elevation of 3194–3226 m. This lakeshore wetland features a typical plateau semi-arid alpine climate with an average annual temperature of -0.7°C (ranging from -31°C to 28°C) and annual precipitation of 322.7 mm. The 12.6 km² sandy area (representing an 8.3% expansion since 2015) exhibits a distinct northwest-to-southeast gradient distribution pattern, transitioning through mobile dune areas, semi-fixed sandy areas, and fixed sandy areas, forming a unique “sand island-wetland” mosaic landscape. Dominant vegetation includes *Poa alpigena* L., *Stipa purpurea* Griseb., *Carex rigescens*, *Leymus secalinus*, *Polygonum sibiricum* L., *Allium przewalskianum*, and *Astragalus adsurgens* Pall., constituting a distinctive alpine wetland plant community.

### 2.2 Samples method and treatment

The sampling site was located in the sandy dune area of Bird Island. Soil samples with consistent *Poa alpigena* L. growth were collected from four 2 m × 2 m quadrats using an S-shaped five-point sampling method. Rhizosphere soil (NG) was collected by vigorously shaking the roots, while bulk soil (NC) was obtained from the 0–20 cm depth surrounding the plants. Post-rainfall rhizosphere soil (YNG) and non-rhizosphere soil (YNC) were collected using the same methodology. According to data from the local hydrological bureau, the Qinghai Lake watershed received heavy rainfall (as classified by the Chinese National Standard “Grade of Precipitation” (GB/T 28592-2012), with 12-hour precipitation reaching 15.0-29.9 mm) on the sampling night. Our post-rainfall sampling was conducted 2 hours after the precipitation ceased. Both rhizosphere and bulk soils were divided into two aliquots. For the first aliquot, liquid nitrogen cryopreservation samples were utilized for DNA extraction and subsequent metagenomics sequencing. Following DNA extraction precipitation and purification procedures, the resulting DNA samples were sent to BGI Technology Co., LTD (Shenzhen, China) for metagenomics sequencing using the BGI-SEQ-500 platform. The second aliquot was used for assessing various soil properties including water content, total nitrogen, total carbon, pH level, and conductivity. The methods or instruments employed for detecting these soil properties are as follows: Soil water content was determined using a JK-100F soil moisture analyzer (Shanghai Jingke Industrial Co., Ltd., China); Total nitrogen was measured with a FOSS Kjeltec 8400 automated Kjeldahl nitrogen analyzer (FOSS Analytical, Denmark); Total carbon was analyzed by a CE-440 elemental analyzer (Exeter Analytical Inc., USA); pH was measured using a pHS-25 benchtop pH meter (Shanghai Leici Instrument Co., China); Electrical conductivity was determined with a DDS-307 conductivity meter (Shanghai Yidian Scientific Instrument Co., Ltd., China).

### 2.3 Data analysis

To obtain high-quality DNA sequences of soil microbial organisms, we employed Trimmomatic software (v3.3) to remove linker sequences. The cleaned linker sequences are provided below.

PrefixPE/1:AAGTCGGAGGCCAAGCGGTCTTAGGAAGACAA;
PrefixPE/2:AAGTCGGATCGTAGCCATGTCGTTCTGTGAGCCAAGGAGTTG.

Subsequently, the following procedures were processed by using the original default parameters unless otherwise specified. According to Megahit (v1.2.9) software default parameters, all read sets were assembled by Megahit software. MetaQUAST (v5.2.0) software was used to evaluate the effects of metagenomics assembly such as the comparison of the assembly results with the reference sequence, the quality of contigs, N50 of the sequences, etc.

Kraken v2 software was used to analyze the comparison of assembly sequences with reference databases including Bacterial, Fungal, Archaebacterial, Protozoan genomes, Viral, and Nt from NCBI. To obtain detailed taxonomic information on species annotation and richness, the taxonomic level of phylum, class, order, family, genus, and species was annotated by combined utilization with Kraken v2 and Bracken. The microorganism α-diversity was analyzed by using Vegan (v2.6-4) software. For the β-diversity index of microorganisms, principal component analysis was performed. To obtain dominant microorganism and their metabolic pathways, LEfSe analysis (LDA score threshold > 2.0) was adopted to analyze raw data of intergroup microorganisms and KEGG pathway enrichment analysis. Raw sequencing was performed on the DNBSEQ™ platform, and quality control of raw reads including adapter trimming and quality filtering was conducted using Trimmomatic (v0.39) with parameters set to SLIDINGWINDOW:4:20 and MINLEN:50. The database of metagenomics sequencing was uploaded to NCBI (https://www.ncbi.nlm.nih.gov/sra/PRJNA867494 and PRJNA1188057). In addition, data processing was performed according to statistical methods by using SPSS 26.0.

## 3 Results

### 3.1 Heterogeneity of physicochemical properties between rhizosphere and bulk soils in Qinghai lake sandy land ecosystem

To gain insights into the heterogeneity of physicochemical indices in the Rhizosphere soil of *Poa alpigena* in the sandy area of Bird Island, Qinghai Lake, the rhizosphere soil (NG), bulk soil (NC), Post-rain rhizosphere soil (YNG) and post-rain bulk soil (YNC) of sandy area, we assessed parameters such as water content, total nitrogen, total carbon, pH, and conductivity (**Table 1**). The results indicate that the total carbon (TC) and total nitrogen (TN) contents in pre-rainfall bulk soils were significantly higher than those in post-rainfall bulk soils (*p-*value < 0.05), whereas the moisture content in both post-rainfall non-rhizosphere and rhizosphere soils showed significant increases compared to pre-rainfall conditions (*p-*value < 0.01).

**Table 1:** Physicochemical properties of rhizosphere and bulk soils in *Poa alpigena* habitats before and after rainfall.

Name	NC	YNC	NG	YNG
Water Content (%)	0.24 ± 0.02b	2.38 ± 0.61a	0.15 ± 0.03b	0.43 ± 0.01b
pH Value	8.91 ± 0.17a	8.99 ± 0.19a	8.59 ± 0.3a	8.71 ± 0.32a
Conductivity ( mS/cm)	0.05 ± 0.01b	0.04 ± 0.001b	0.06 ± 0.02ab	0.08 ± 0.02a
Total carbon (g/Kg)	5.12 ± 0.4a	2.59 ± 0.35b	5.14 ± 0.45a	2.63 ± 0.35b
Total nilrogen (g/Kg)	0.42 ± 0.05a	0.20 ± 0.05b	0.26 ± 0.03b	0.23 ± 0.07b

Different superscript letters denote significant differences between quadrats (*p*-Value < 0.05).

### 3.2 Metagenomic sequencing reveals differential responses of soil microbial communities to precipitation in *Poa alpigena*

Metagenomic sequencing results indicated that for each sample, with over 10 GB of sequence data, an average of more than 36 million paired sequences were obtained. These data demonstrate a stable sequencing depth of the microbiota. Additionally, 23%–58% of the sequenced reads could not be matched to known taxa in existing reference databases, which may reflect either the presence of novel microbial lineages or limitations in current database coverage of alpine sandy soil microbiomes (**Table 2**). The percentage of microbial sequences in non-rhizosphere soil after rain ranged from 21.31% to 25.15%, with bacteria accounting for 20.25%. In rhizosphere soil after rain, the percentage of microbial sequences ranged from 23.56% to 24.52%, both significantly lower than in pre-rain soil, where bacteria reached 21.01%, higher than in non-rhizosphere soil. Before rain, fungi, viruses, and protozoa accounted for 1.42%, 0.16%, and 0.18% of the total sequences in rhizosphere and bulk soils, respectively. After rain, these proportions decreased to 0.40%, 0.04%, and 0.04%, showing a significant difference (*p-*value < 0.05).

**Table 2:** Sequencing results of soil microorganisms in *Poa alpigena* sandy land of Bird Island, Qinghai Lake before and after rainfall.

Name	NC	YNC	NG	YNG
Number of raw reads	36702067.5 ± 1074957.9a	38388179.3 ± 14953.4a	36367431.5 ± 2157852.8a	38183447.3 ± 431801.8a
Classified reads(%)	42.88 ± 0.72b	24.85 ± 1.97c	45.28 ± 1.55a	25.74 ± 0.47c
Chordate reads(%)	6.23 ± 0.06a	1.57 ± 0.14b	6.20 ± 0.27a	1.65 ± 0.02b
Unclassified reads(%)	57.13 ± 0.72b	75.18 ± 1.97a	54.72 ± 1.55c	74.27 ± 0.47a
Microbial reads(%)	36.50 ± 0.67c	23.23 ± 1.92b	38.94 ± 1.80a	24.04 ± 0.48c
Bacterial reads(%)	28.80 ± 0.54c	20.25 ± 1.87b	31.22 ± 1.91a	21.01 ± 0.46c
Viral reads(%)	0.16 ± 0.00a	0.04 ± 0.00b	0.16 ± 0.01a	0.04 ± 0.01b
Fungal reads(%)	1.42 ± 0.04a	0.40 ± 0.02b	1.42 ± 0.06a	0.43 ± 0.01b
Protozoan reads(%)	0.18 ± 0.00a	0.04 ± 0.01b	0.18 ± 0.01a	0.04 ± 0.00b

### 3.3 Rainfall significantly reduces soil bacterial alpha diversity, particularly in non-rhizosphere microbiota

**Figure 1:**
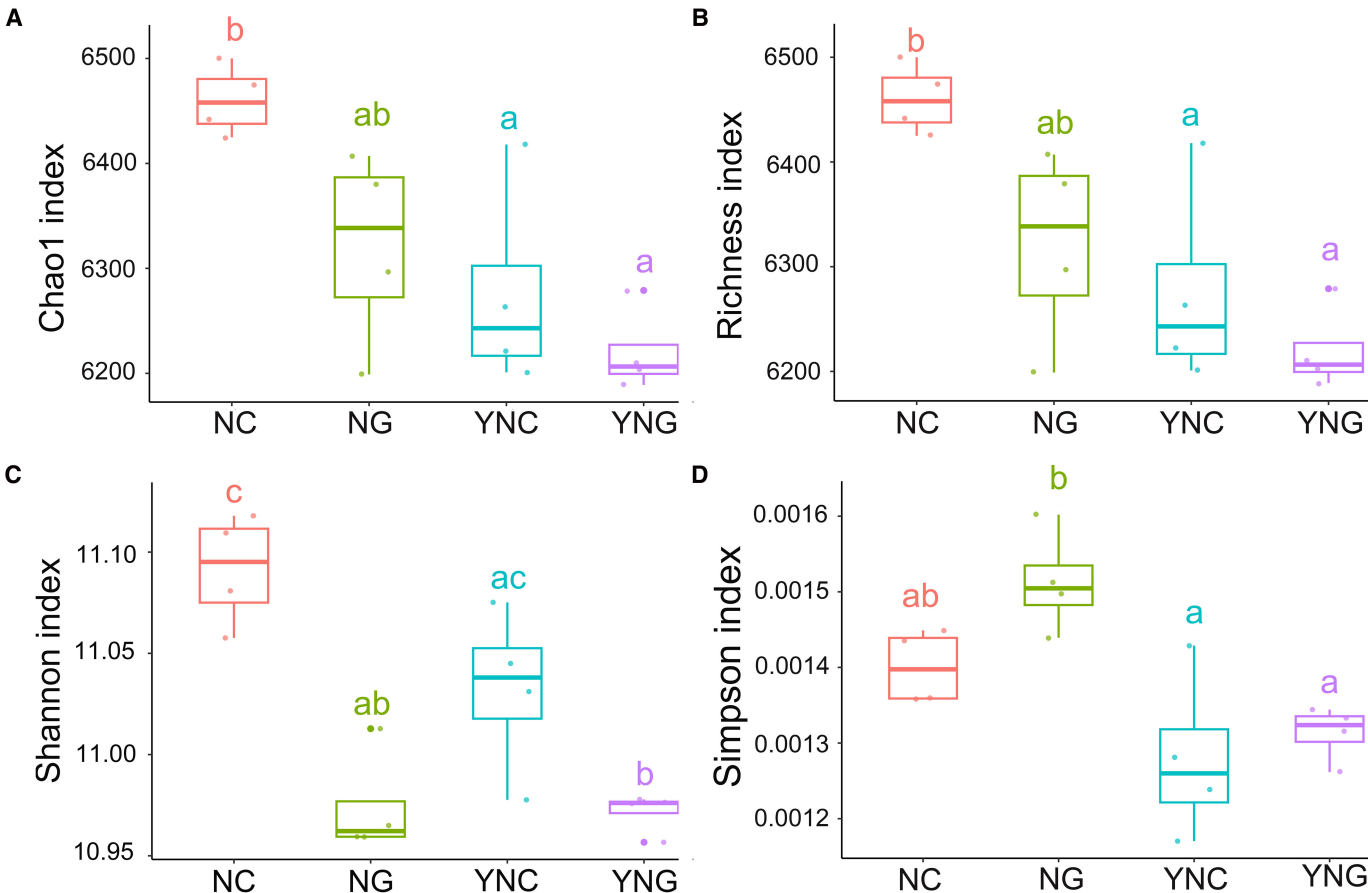
Pre- and post-rainfall α-diversity of rhizosphere and bulk soil microbial communities. **(A)** Chao 1 index, **(B)** Richness index, **(C)** Shannon index, and **(D)** Simpson index. Letters shared across boxplots indicate no significant difference, while distinct letters denote significant differences.

Alpha diversity is used to characterize the microbial community diversity of individual samples, reflecting both species richness and diversity levels. In this study, the Chao1 index and Richness index were employed to assess microbial community richness, while the Shannon index and Simpson index were used to evaluate community diversity. Boxplots visually illustrate whether intergroup differences in species diversity were significant. The impact of precipitation changes on microbial community diversity revealed that pre-rainfall non-rhizosphere (NC) samples exhibited significantly higher Chao1 and Richness indices compared to other conditions (*p-*value < 0.05). In contrast, post-rainfall non-rhizosphere (YNC) samples showed a notable decline in Chao1 and Richness, indicating that rainfall reduces soil microbial abundance. Furthermore, rhizosphere microbial richness followed the trend NC > YNC, while non-rhizosphere microbial richness followed NG > YNG. The Shannon index was highest in NC and lowest in YNG. NC’s Shannon index was significantly higher than YNC’s (*p-*value < 0.05), whereas the difference between NG and YNG was not significant (*p-*value ≥ 0.05). The Simpson index exhibited substantial variation, decreasing progressively from pre- to post-rainfall conditions, with NG showing the highest value. This further demonstrates that post-rainfall rhizosphere microbial diversity was significantly constrained. NG’s Simpson index was significantly higher than all other conditions, and the difference between NG and YNG was statistically significant (*p*-value < 0.05) (**Figure 1**). In summary, rainfall has a substantial impact on soil bacterial alpha diversity, particularly in reducing microbial richness and altering diversity patterns in both rhizosphere and non-rhizosphere communities.

### 3.4 Rainfall drives the restructuring of microbial community structure and function in rhizosphere and bulk soils of desert ecosystems

Precipitation is a key limiting factor in desert ecosystem dynamics. Variations in rainfall not only alter soil moisture and organic matter content to influence microbial activity, but also modify plant community biomass and coverage, thereby shaping soil microbial composition (Yang et al., 2021). This study employed high-throughput sequencing to systematically analyze compositional changes in soil microbial communities at both phylum and genus levels, comparing rhizosphere (NG/YNG) and non-rhizosphere (NC/YNC) soils before and after precipitation events.

**Figure 2:**
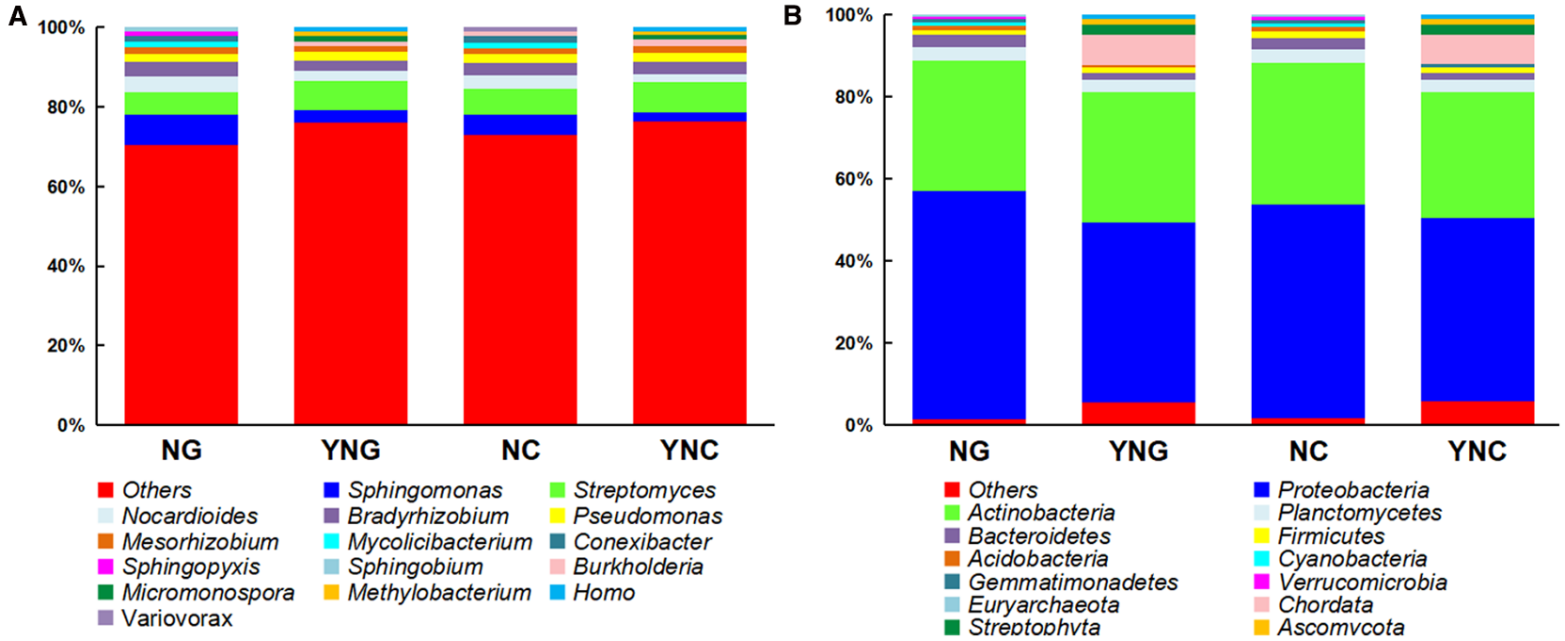
Microbial relative abundance at the phylum **(A)** and genus **(B)** taxonomic levels across NC, YNC, NG, and YNG groups.

At the phylum level, significant niche differentiation was observed (**Figure 2A**; **Supplementary Table S1**). High-throughput sequencing revealed Proteobacteria, Actinobacteria, and Planctomycetes as the dominant phyla (mean relative abundance >28.11%) in both rhizosphere and bulk soils before and after rainfall events. Notably, Proteobacteria exhibited the highest mean relative abundance (49.10%). Specifically, pre-rainfall samples showed significantly different Proteobacteria abundance between rhizosphere (NG: 55.63%) and bulk soils (NC: 51.92%) (*p*-value < 0.05), suggesting that rhizodeposits (e.g., organic acids) may preferentially stimulate Proteobacteria growth in the rhizosphere microenvironment (Keiluweit et al., 2015). Post-rainfall analysis revealed a sharp 11.40% decline in Proteobacteria abundance within the rhizosphere (*p*-value < 0.05), while bulk soils maintained relative stability (51.92% to 44.84%). This differential response suggests that hydrological leaching preferentially removes rhizospheric microbial populations, whereas the environmentally buffered non-rhizosphere zone demonstrates greater community resilience (Bai et al., 2014). Notably, Actinobacteria populations maintained exceptional stability, showing no statistically significant differences in relative abundance across both rhizosphere (NG: 31.56% ± 0.42; YNG: 31.65% ± 0.38) and bulk soils (NC: 34.62% ± 0.51; YNC: 30.60% ± 0.47) following precipitation events (*p*-value = 0.82, ANOVA). This pronounced resistance to hydrological disturbance strongly indicates that Gram-positive bacterial phyla, particularly Actinobacteria and Acidobacteria, possess intrinsic physiological adaptations that confer remarkable insensitivity to soil moisture dynamics (Solans et al., 2021).

At the genus-level resolution, our analysis revealed distinct ecological adaptation strategies among soil microbiota (**Figure 2B**; **Supplementary Table S1**). Most strikingly, Streptomyces populations demonstrated significant rainfall-responsive proliferation in bulk soils, with relative abundance increasing from 6.43 ± 0.21% (NC) to 7.56 ± 0.18% (YNC) (*p*-value = 0.032, t-test). This moisture-dependent activation likely reflects the upregulation of its dual functional capacities: (1) extracellular enzyme systems for complex organic matter mineralization, and (2) antimicrobial secondary metabolite biosynthesis (Aida et al., 2018). In striking contrast, Sphingomonas populations exhibited significant declines across both rhizosphere and bulk soil compartments (decreasing by 4.55% and 2.73%, respectively; *p*-value < 0.05). Particularly noteworthy was Pseudomonas, which maintained stable colonization rates despite environmental fluctuations (rhizosphere: 2.00-2.11%; bulk soil: 2.31-2.32%; *p*-value > 0.05). This ecological persistence suggests its siderophore-producing capacity may represent an evolutionary adaptation for environmental stress resilience through specialized metabolic regulation (Wang et al., 2024). In summary, rainfall significantly reshapes the microbial community structure and function in Qinghai Lake’s Bird Island Wetland by modulating soil moisture dynamics and physicochemical properties. Future studies should combine multi-omics approaches with continuous environmental monitoring to elucidate the functional gene expression of key microbial taxa and evaluate long-term shifts in their ecological roles. Such insights will offer a scientific foundation for conserving and restoring high-altitude wetland ecosystems.

### 3.5 LEfSe analysis reveals rainfall-induced shifts in dominant microbial taxa between rhizosphere and bulk soils

**Figure 3:**
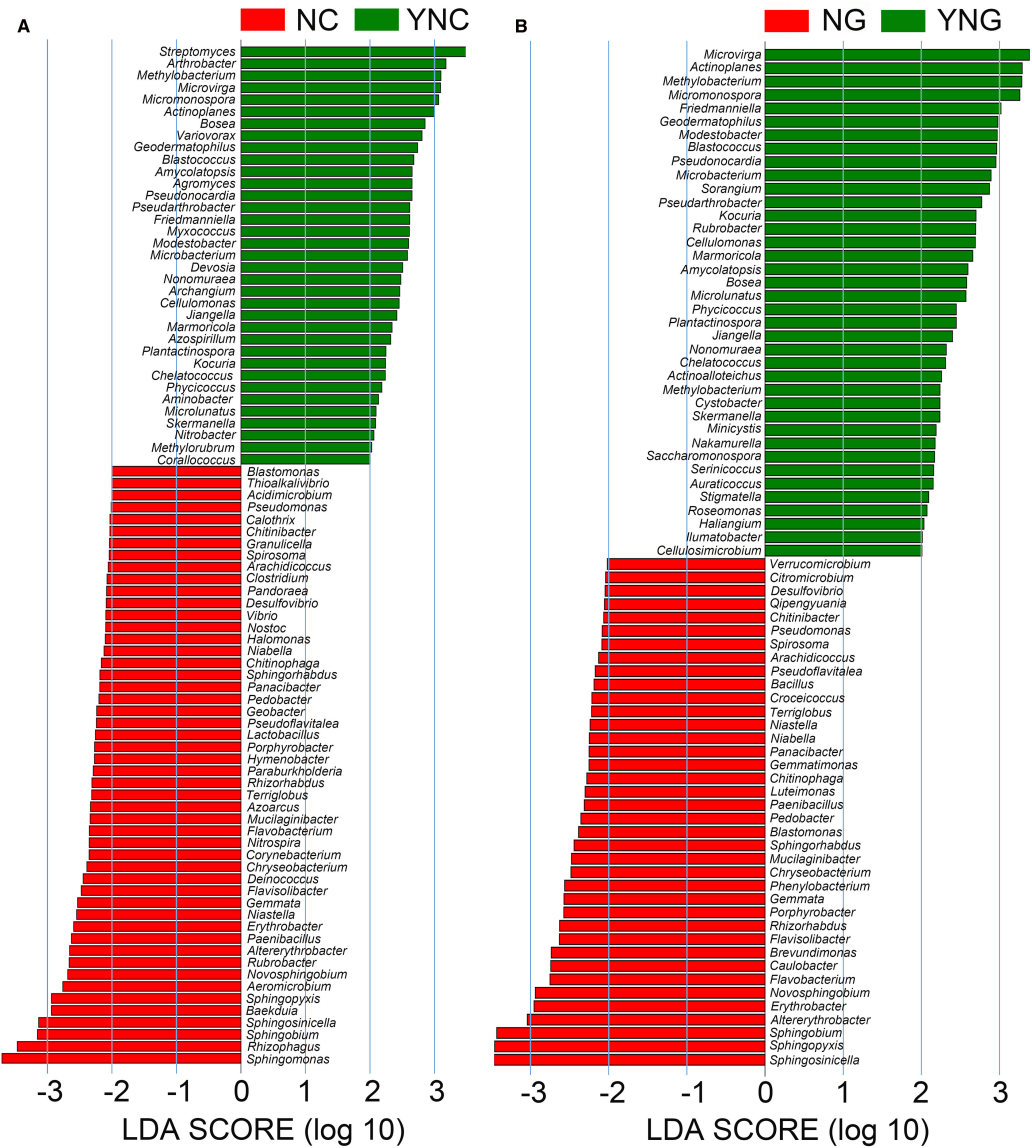
Differentially abundant microorganisms between rhizosphere and bulk soils post precipitation (NC vs YNC, NG vs YNG). In **A** and **B**, the green bars represent microbial species with significant abundance differences in non-rhizosphere soil and rhizosphere soil of Bird Island after rain, while the red bars represent microbial species with significant abundance differences in non-rhizosphere soil and rhizosphere soil of Bird Island before rain. The longer the bar, the higher the significance of the difference in the corresponding microorganism.

To characterize rainfall-induced changes in microbial communities, we conducted Linear Discriminant Analysis Effect Size (LEfSe) to identify differentially abundant taxa. Significant compositional shifts were observed in non-rhizosphere soil microbiota following rainfall events (**Figure 3**). A total of 57 microbial genera showed differential abundance (LDA score > 2), with 25 taxa enriched under pre-rainfall conditions (NC group) and 32 taxa dominating post-rainfall soils (YNC group). Pre-rainfall bulk soils were primarily characterized by taxa often associated with drier conditions, including *Geobacter*, *Lactobacillus*, *Thioalkalivibrio*, *Calothrix*, and *Pseudomonas*. In contrast, post-rainfall conditions favored taxa frequently linked to higher moisture availability such as *Azospirillum*, *Methylobacterium*, *Nitrospira*, and *Variovorax*, suggesting a hydrological influence on microbial community succession.

LEfSe analysis of rhizosphere samples identified 61 differentially abundant taxa (LDA > 2), with a clear separation between pre-rainfall (NG, 23 taxa) and post-rainfall (YNG, 38 taxa) communities. The rhizosphere prior to rainfall was enriched with taxa such as *Verrucomicrobium*, *Citromicrobium*, *Qipengyuania*, and *Sphingobium*, which have been reported in drought-adapted contexts. After rainfall, moisture-favored taxa including *Cellulomonas*, *Methylobacterium*, and *Actinoplanes* became more abundant, indicating a hydrologically driven shift in community composition.

The comparative analysis revealed that microbial communities underwent substantial restructuring in response to rainfall. Pre-rainfall conditions, characterized by low soil moisture and oxidative stress, were dominated by drought-resistant genera such as Geobacter and Pseudomonas, which may employ stress tolerance strategies including phytohormone biosynthesis (Fortune et al., 2020; Virginia et al., 2024). Increased soil moisture after rainfall likely created localized anaerobic conditions, supporting the growth of facultative anaerobes (e.g., Desulfovibrio, Vibrio) and bacteria involved in nitrogen cycling (e.g., Nitrospira) (Liu et al., 2024).

### 3.6 Differential responses of soil microbial metabolism to rainfall and rhizosphere effects revealed by KEGG pathway analysis

**Figure 4:**
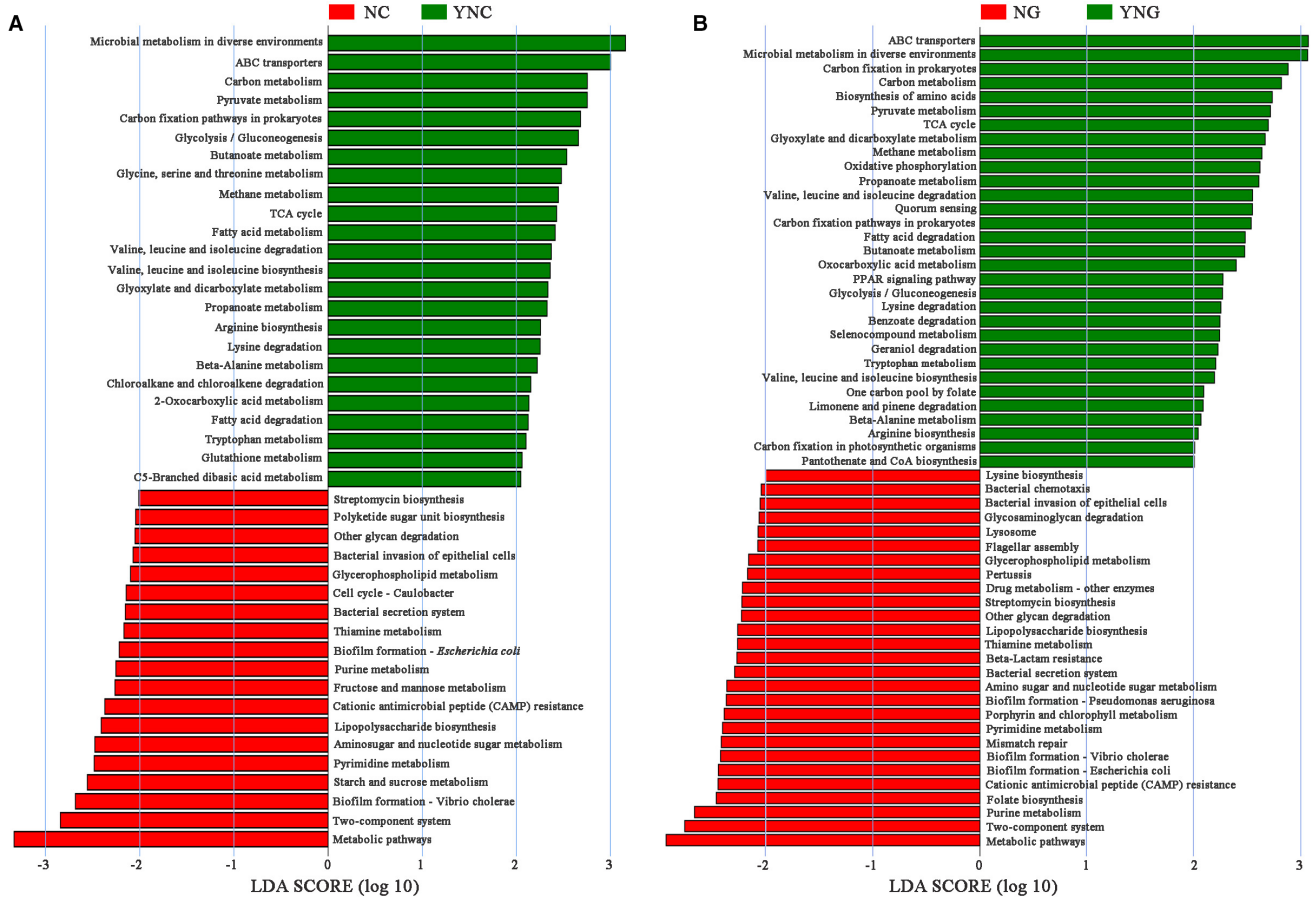
The soil microbial metabolic pathways by LEfSe analysis (NC vs YNC, NG vs YNG). In **A** and **B**, the green bars represent metabolic pathways with differences in non-rhizosphere soil and rhizosphere soil of Bird Island after rain, while the red bars represent metabolic pathways with differences in non-rhizosphere soil and rhizosphere soil of Bird Island before rain. The longer the bar, the higher the significance of the difference in the corresponding metabolic pathway.

KEGG pathway enrichment analysis comparing four soil regimes—NC (pre-rainfall non-rhizosphere), YNC (post-rainfall non-rhizosphere), NG (pre-rainfall rhizosphere), and YNG (post-rainfall rhizosphere)—demonstrated that both precipitation events and rhizosphere presence significantly altered microbial metabolic potential (**Figure 4**). In bulk soils, rainfall induced a metabolic shift from carbohydrate catabolism to energy and nutrient acquisition strategies. Pre-rainfall conditions (NC) were characterized by enriched starch/sucrose metabolism (ko00500), pyrimidine metabolism (ko00240), and aminosugar/nucleotide sugar metabolism (ko00520), while post-rainfall samples (YNC) showed increased methane metabolism (ko00680), prokaryotic carbon fixation (ko00720), and ABC transporter activity (ko02010). The rhizosphere microbiome exhibited even greater metabolic plasticity, with 58 differentially abundant pathways (LDA>2) between rainfall conditions. Pre-rainfall rhizosphere soils (NG) uniquely upregulated virulence-associated pathways including E. coli biofilm formation (ko02026), V. cholerae biofilm formation (ko05111), and cationic antimicrobial peptide resistance (ko01503). In contrast, post-rainfall rhizosphere communities (YNG) shifted toward metabolic processes typically associated with increased water availability and plant-microbe interactions.

### 3.7 Analysis of soil microbial correlations and their relationship with soil physicochemical properties before and after rainfall events

**Figure 5:**
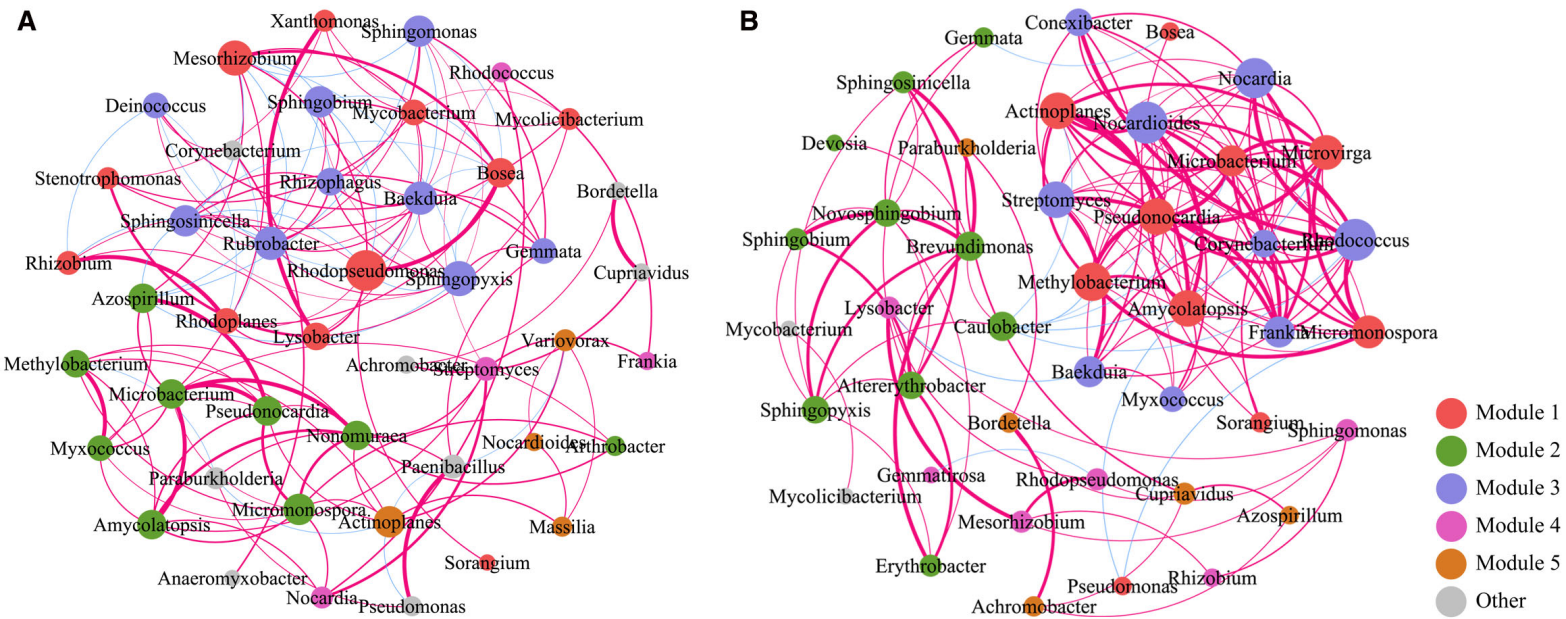
Correlation analysis of non-rhizosphere **(A)** and rhizosphere **(B)** soil microorganisms before and after rain.

To investigate the interrelationships of soil microbial abundance in Qinghai Lake Bird Island Wetland before and after rainfall, we conducted correlation analyses of soil microbial communities. As illustrated in the figure, each node represents a distinct microorganism, with node colors corresponding to different modules and sizes reflecting relative abundance. Red and blue connecting lines indicate significant positive and negative correlations between microbial taxa, respectively, with line thickness representing the absolute magnitude of correlation coefficients. In bulk soils (**Figure 5A**), the core microbial taxa comprised 18 genera, primarily ecologically functional groups such as *Mesorhizobium*, *Sphingobium*, *Sphingomonas*, *Rhodopseudomonas*, *Bosea*, and *Mycolicibacterium*. Notably, *Microbacterium* exhibited strong positive correlations (*p*-value < 0.05) with *Pseudomonas*, *Nonomuraea*, and *Amycolatopsis*, while *Mesorhizobium* showed significant negative correlations (*p-value* < 0.05) with *Sphingomonas* and *Sphingobium*. These robust positive correlations (*p-value* < 0.05) suggest the formation of functionally complementary metabolic networks among these taxa, enabling coordinated responses to rainfall-induced environmental shifts. Conversely, the antagonistic relationship between *Mesorhizobium* and *sphingomonads* (*Sphingomonas*, *Sphingobium*) may reflect resource competition or niche exclusion in non-rhizosphere environments.

Network analysis of rhizosphere soils revealed inter-genus correlations among 41 microbial taxa (**Figure 5B**), including *Nocardia*, *Paraburkholderia*, *Streptomyces*, *Mesorhizobium*, *Actinoplanes*, *Methylobacterium*, *Caulobacter*, *Pseudonocardia*, and *Amycolatopsis*. Significant positive correlations (*p*-value < 0.05, FDR-corrected) were observed between Pseudonocardia and both Methylobacterium and actinomycetes (Actinoplanes/Amycolatopsis), indicating rainfall-mediated co-occurrence patterns. These interactions suggest the coexistence of three functional guilds: decomposers (Streptomyces/Pseudonocardia), methylotrophs (Methylobacterium), and plant symbionts (Mesorhizobium) in the rhizosphere.

**Figure 6:**
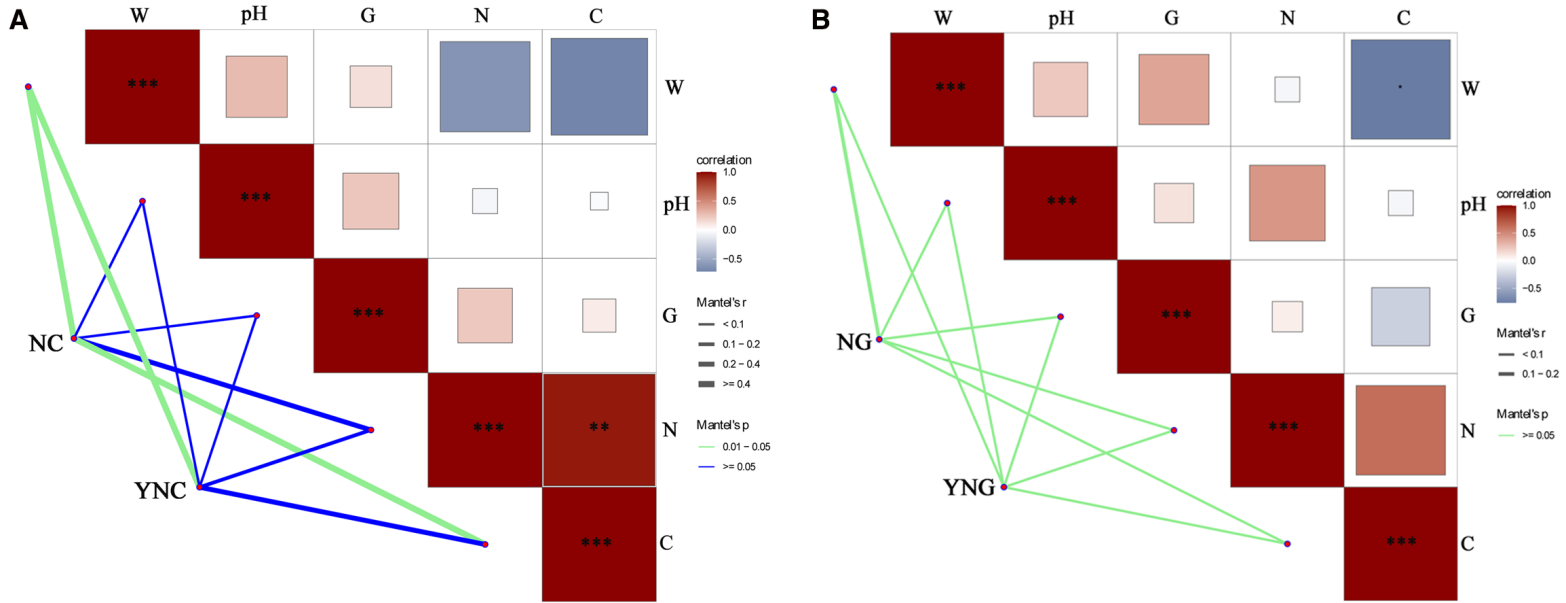
Analysis of soil microbial communities and physicochemical properties in bulk **(A)** and rhizosphere **(B)** soils pre- and post-rainfall. **p<0.01; ***p<0.001.

The figure displays correlation analysis between soil microbial communities and physicochemical properties in Bird Island Wetland. In bulk soils (**Figure 6A**), pre-rainfall microbiota showed significant associations with soil total carbon (TC) and moisture content (SWC) (*p*-value < 0.05), but not with total nitrogen (TN), pH or electrical conductivity (EC) (*p*-value > 0.05). Post-rainfall communities were predominantly correlated with SWC (*p*-value < 0.05), while maintaining no significant relationships with TC, TN, pH or EC (*p*-value > 0.05), indicating that soil moisture dynamics serve as the key determinant of microbial community variations in non-rhizosphere environments (Tian et al., 2024). In the rhizosphere sampling sites (**Figure 6B**), microbial communities showed limited responsiveness to soil physicochemical properties both before and after rainfall events. However, significant differences were observed between non-rhizosphere and rhizosphere soils in their enriched microbial communities following precipitation, demonstrating that these variations are closely associated with plant rhizosphere effects. The study by Toledo et al. also found that the physicochemical properties of rhizosphere and bulk soils respond to changes in water content (Toledo et al., 2023).

## 4 Discussion

### 4.1 Analysis of dominant soil microbial abundance before and after rainfall

Rainfall reduced soil microbial diversity in Poa alpigena grasslands on the Qinghai-Tibetan Plateau, potentially linked to a significant post-rain rise in soil water content within non-rhizosphere zones. While moderate wetness generally enhances microbial growth, waterlogging or excessively high water content suppresses microbial activity. This underscores the critical role of soil moisture thresholds in sustaining microbial diversity. Altered precipitation regimes reshape soil nutrient mineralization by modulating water content, pH, and nutrient availability, consequently shifting microbial community composition. Meta-genomic sequencing of Poa alpigena rhizosphere microbiota from Qinghai Lake’s Bird Island demonstrated that bacterial read counts significantly surpassed fungal reads. Post-rainfall, bacterial relative abundance rose by 12.5%, substantially exceeding the 4.8% increase in fungi. These results corroborate implying that bacterial communities respond more acutely to precipitation variability, possibly attributable to faster ribosome biosynthesis and superior dispersal capacity (Yu et al., 2024). Notably, in desert steppe ecosystems, Actinobacteria, Proteobacteria, and Acidobacteria are the dominant bacterial phyla (Liu, 2021). Among these, Actinobacteria sustain higher abundance under fluctuating precipitation through adaptive strategies such as increased cell wall thickness (Manzoni et al., 2012). Nevertheless, microbial diversity demonstrates a characteristically delayed response to precipitation variability, a phenomenon potentially mediated by inherent community stability mechanisms. More strikingly, the extreme low-temperature and hypoxic conditions endemic to alpine meadow ecosystems confer a competitive advantage to Firmicutes, whose ecological dominance is facilitated by their evolutionarily conserved spore-forming capability (Zhao et al., 2024). This characteristic was further validated through comparative analysis with Antarctic strain *Sporosarcina* sp. Lc50-2 (99.87% similarity) (Zhao et al., 2024). These results indicate that the strain’s adaptive evolution is intrinsically linked to cryohumid environments. These findings establish a critical framework for elucidating microbial community response mechanisms to climate change dynamics.

### 4.2 Divergent metabolic pathway responses to precipitation changes in rhizosphere versus non-rhizosphere soil microbial communities

This study shows that changes in rainfall patterns trigger distinct metabolic responses in soil microbial communities. Before rain, bulk soils were enriched in pathways for mobilizing carbohydrate reserves—such as starch and sucrose metabolism—and for synthesizing nucleotide precursors, including pyrimidine and amino sugar metabolism. After rain, soils showed increased activity in anaerobic energy production through methane metabolism, inorganic carbon assimilation via prokaryotic carbon fixation, and substrate uptake systems like ABC transporters. Together, these findings suggest that drought stress selects microbes capable of coordinating three key survival strategies: breaking down intracellular carbon stores first, producing osmoregulatory solutes, and maintaining genomic stability. These mechanisms align with the stress adaptation model proposed by Zhang and colleagues (Zhang et al., 2025). The post-rain metabolic shift likely arises from precipitation-driven hydration dynamics that promote localized anaerobiosis while activating CO_2_ assimilation pathways. The activation of carbon fixation pathways likely results from post-rainfall depletion of labile organic carbon through leaching or accelerated decomposition, driving microbial communities to enhance carbon use efficiency as soil organic carbon availability declines (Shi et al., 2024). Notably, post-rainfall soils exhibited significant upregulation of glycine, serine, and threonine metabolic pathways (p<0.05), potentially linked to accelerated amino acid turnover rates mediated by microbial reactivation (Amino Acids, 2019). Rhizosphere soil microbiomes exhibited distinct metabolic reprogramming before and after precipitation events. Specifically, non-irrigated groups (NG) showed significant enrichment of biofilm formation pathways (e.g., Escherichia coli and Vibrio cholerae) and cationic antimicrobial peptide resistance, particularly in Enterobacteriaceae-associated pathways. These findings suggest that drought conditions selectively favor the colonization and interspecies interactions of stress-adapted microbiota, where biofilm-mediated niche construction enhances rhizosphere environmental adaptation. Such cooperative microbial behaviors likely play a key role in shaping plant-associated microbiome assembly and holobiont fitness (Amino Acids, 2019). In contrast, rhizosphere soil collected after rainfall (YNG) showed marked enrichment in fatty acid metabolism and pathways associated with the degradation and biosynthesis of valine, leucine, and isoleucine. Strikingly, aspartate, lysine, threonine, leucine, isoleucine, and valine levels all shifted dynamically in response to precipitation. These amino acids are metabolically linked: lysine, threonine, and isoleucine are synthesized through aspartate-derived branching pathways, while leucine, isoleucine, and valine form the group of branched-chain amino acids (BCAAs), underscoring their coordinated roles in rain-induced metabolic adaptation (Azevedo et al., 1997). These findings demonstrate the metabolic plasticity of soil microbiota in adapting to post-precipitation resource fluctuations and environmental stresses through dynamic equilibrium between catabolic and anabolic processes, enabling responses to complex ecological demands. Notably, both bacterial secretion systems and two-component systems (TCSs) showed sustained activation in the rhizosphere. This observation aligns with the known characteristics of TCSs as sophisticated regulatory networks that not only facilitate inter-system signal transduction via sensor-response protein interactions but also indirectly modulate metabolic pathways including amino acid metabolism. These results collectively highlight the critical role of microbe-plant signaling communication in rhizosphere ecological adaptation (Torres and Müller, 2025).

Comparative analysis revealed distinct ecological strategies between rhizosphere and non-rhizosphere microbiomes. Non-rhizosphere microbiota prioritized core energy production (TCA cycle, oxidative phosphorylation) and environmental adaptation, while rhizosphere communities specialized in plant-microbe interactions via secondary metabolite biosynthesis and pathogenesis pathways. This niche-specific specialization shows non-rhizosphere microbes responding to abiotic stresses versus rhizosphere communities engaging in host interactions. Crucially, both systems maintained essential ABC transporter and carbon metabolic functions, underscoring their universal importance across soil environments.

### 4.3 Temporal dynamics of soil microbial communities and their associations with physicochemical properties across precipitation events

This study uncovers the intricate interactions among soil microorganisms in Qinghai Lake’s Bird Island wetland during rainfall events. In bulk soil, Microbacterium and Pseudomonas exhibited a significant positive correlation (*p*-value < 0.05), likely driven by functional synergy. Notably, Pseudomonas possesses phytase activity and multiple plant growth-promoting (PGP) traits, including organic/inorganic phosphorus solubilization, indole-3-acetic acid (IAA) secretion, nitrogen fixation, and phytopathogen antagonism (Walker, 2004). Moreover, Microbacterium can produce plant growth-promoting volatile organic compounds (VOCs), exhibit strong phosphate-solubilizing and nitrogen-fixing activities, and synthesize indole-3-acetic acid (IAA). The synergistic effects of these traits may collectively enhance plant nutrient utilization efficiency (Moturu et al., 2023). In the rhizosphere soil, the strong positive correlation (*p*-value < 0.05) between Pseudonocardia and Methylobacterium may form a functional symbiotic module. Pseudonocardia can produce antibiotics and exhibits potent bactericidal properties (Song et al., 2018). Moreover, these microorganisms can degrade various recalcitrant compounds, thereby supplying carbon sources to Methylobacterium and stimulating its growth. In return, Methylobacterium facilitates host plant growth through the synthesis of IAA, ammonia, siderophores, ACC deaminase, cellulase, and catalase. This “degradation-metabolism-symbiosis” cascade demonstrates remarkable consistency with the theoretical framework of dynamic microbial interaction networks in the rhizosphere (Watsana et al., 2018; Chowdhury et al., 2025). The results suggest that rainfall events may activate specific microbial functional consortia by altering water availability.

Our findings demonstrate that soil water content serves as the primary driver of microbial community restructuring in bulk soil (*p* < 0.05). Intriguingly, rhizosphere microbial communities showed minimal responsiveness to soil physicochemical properties (*p* > 0.05), likely due to the predominant influence of root exudates in stabilizing the rhizosphere environment. These biological regulatory processes may effectively mask the direct impacts of abiotic soil parameters.

This study provides clear evidence that rainfall events induce a substantial reduction in bacterial alpha diversity and drive structural reorganization of microbial communities in the sandy alpine ecosystem of Qinghai Lake. These effects are more pronounced in bulk soil compared to the rhizosphere, highlighting the role of plant-root mediation in mitigating environmental disturbances. Mechanistically, the shifts are attributed to moisture-induced changes in taxonomic composition—particularly a marked decrease in Proteobacteria and the consistent stability of Actinobacteria—along with post-rainfall metabolic transitions toward anaerobic respiration and carbon fixation pathways. The buffering capacity of the rhizosphere, facilitated by root exudates, underlies enhanced microbial structural stability and functional resilience under fluctuating moisture conditions. To advance these insights, future studies should employ integrated multi-omics methodologies and controlled field experiments to quantitatively track exudate flux and elucidate the genetic determinants of microbial adaptation to drought stress.

## 5 Conclusion

This research elucidates the differential responses of rhizosphere and bulk soil microbial communities to precipitation events in the sandy soil of Qinghai Lake Basin, underscoring their pivotal role in maintaining ecosystem stability. Our results demonstrate that rainfall induces a significant reduction in bacterial alpha diversity, with bulk soils exhibiting greater sensitivity than their rhizosphere counterparts. Concurrently, precipitation drives substantial restructuring of microbial community composition and metabolic functions. Taxonomic analysis revealed pronounced niche differentiation among key bacterial phyla: Proteobacteria populations declined markedly in the rhizosphere following rainfall, likely due to hydrological leaching, whereas Actinobacteria displayed exceptional environmental resilience. Metabolically, microbial communities shifted from carbohydrate-dominated catabolism under arid conditions to anaerobic energy production and carbon fixation pathways post-precipitation, highlighting their remarkable adaptive capacity to moisture fluctuations. The rhizosphere microbiome exhibited distinct functional specialization, including drought-induced biofilm formation and rainfall-enhanced amino acid metabolism, reflecting tight plant-microbe interactions. Correlation analyses identified soil moisture and total carbon as the primary determinant of microbial dynamics in bulk soils, unlike bulk soils, rhizosphere microbial assemblages exhibited negligible sensitivity to variations in soil elemental content, maintaining stability across pre- and post-rainfall conditions. These findings provide novel insights into the mechanisms underlying alpine ecosystem responses to climatic variability and offer a scientific foundation for ecological restoration of degraded sandy lands. Future investigations should employ integrated multi-omics approaches to delineate the functional genetic basis of microbial-mediated ecosystem resilience under variable precipitation scenarios.

## Data Availability

The datasets presented in this study can be found in online repositories. The names of the repository/repositories and accession number(s) can be found in the article/**Supplementary Material**.
